# Multi-Institutional Study of Dorsal Onlay Urethroplasty of the Membranous Urethra after Endoscopic Prostate Procedures: Operative Results, Continence, Erectile Function and Patient Reported Outcomes

**DOI:** 10.3390/jcm10173969

**Published:** 2021-09-02

**Authors:** Javier C. Angulo, Juan F. Dorado, Connor G. Policastro, Francisco E. Martins, Keith Rourke, Erick A. Ramírez, Jay Simhan, Eric S. Li, Paul Rusilko, Krishnan Venkatesan, Jonathan N. Warner, Jaime Gago, Ignacio Arance, Dmitriy Nikolavsky

**Affiliations:** 1 Department of Medical Clinic, Faculty of Biomedical Sciences, Universidad Europea de Madrid, 28905 Madrid, Spain; jfdorado@pertica.es (J.F.D.); jgago5393@gmail.com (J.G.); ignacioarance@gmail.com (I.A.); 2 PeRTICA Statistical Solutions, 28906 Madrid, Spain; 3 Department of Urology, SUNY Upstate Medical University, Syracuse, NY 13210, USA; cgpolicastro@gmail.com (C.G.P.); dnikolavsky@hotmail.com (D.N.); 4 Centro Hospitalar Universitário de Lisboa Norte, Hospital Santa María, 1649-035 Lisboa, Portugal; faemartins@gmail.com; 5 Department of Urology, Alberta University, Edmonton, AB T6G 2R3, Canada; krourke@ualberta.ca; 6 Hospital Angeles Mocel, Mexico City 11850, Mexico; dr_erick08uro@hotmail.com; 7 Department of Urology, Einstein Medical Center/Fox Chase Cancer Center, Philadelphia, PA 19111, USA; Jay.Simhan@fccc.edu (J.S.); lieric00@einstein.edu (E.S.L.); 8 Department of Urology, University of Pittsburgh, Pittsburgh, PA 15260, USA; prusilko19@gmail.com; 9 Urology Department, Georgetown University, Washington, DC 20057, USA; krishnan38@gmail.com; 10 City of Hope Comprehensive Cancer Center, Los Angeles, CA 91010, USA; jonathan.n.warner@gmail.com

**Keywords:** urethral stenosis, transurethral resection of the prostate, buccal mucosa graft, dorsal onlay, urethroplasty, continence, erection, outcomes

## Abstract

(1) Background: To critically evaluate dorsal onlay buccal mucosal graft urethroplasty (DOBMGU) for posterior urethral stenosis repair following transurethral resection and other endoscopic prostate procedures. (2) Methods: A retrospective multi-institutional review of patients with membranous or bulbomembranous urethral stenosis for whom treatment with DOBMGU was conducted after receipt of prostate endoscopic procedures. Baseline data, peri-operative care, post-operative care and patient-reported outcomes were analyzed. The primary outcomes were procedural failure and development of de novo stress urinary incontinence (SUI). The secondary outcomes were changes in voiding, sexual function and patient satisfaction. (3) Results: A total of 107 men with a mean age of 69 ± 9.5 years and stenosis length of 3.5 ± 1.8 cm were included. Prior endoscopic procedures among participants were 47 patients (44%) with monopolar TURP, 33 (30.8%) with bipolar TURP, 16 (15%) with Greenlight laser, 9 (8.4%) with Holmium laser enucleation and 2 (1.9%) with bladder neck incision. At a mean follow-up time of 59.3 ± 45.1 months, stenosis recurred in 10 patients (9.35%). Multivariate analysis confirmed that postoperative complications (OR 12.5; *p* = 0.009), history of radiation (OR 8.3; *p* = 0.016) and ≥2 dilatations before urethroplasty (OR 8.3; *p* = 0.032) were independent predictors of recurrence. Only one patient (0.9%) developed de novo SUI. Patients experienced significant improvement in PVR (128 to 60 cc; *p* = 0.001), Uroflow (6.2 to 16.8 cc/s; *p* = 0.001), SHIM (11.5 to 11.7; *p* = 0.028), IPSS (20 to 7.7; *p* < 0.001) and QoL (4.4 to 1.7; *p* < 0.001), and 87 cases (81.3%) reported a GRA of + 2 or better. (4) Conclusions: DOBMGU is an effective and safe option for patients with posterior urethral stenosis following TURP and other prostate endoscopic procedures. This non-transecting approach minimizes external urinary sphincter manipulation, thus limiting postoperative risk of SUI or erectile dysfunction.

## 1. Introduction

Traditionally, transurethral resection of the prostate (TURP) has been the gold standard surgical treatment for lower urinary tract symptoms due to benign prostatic obstruction (BPO) [1,2]. More recently, other endoscopic approaches, such as KTP laser vaporization and Ho:YAG laser enucleation of the prostate (HoLEP), are proving to be good alternatives to TURP, and transurethral incision of the prostate remains a valid option in the case of small prostate size without a median lobe [3].

None of these approaches are devoid of complications, and the development of urethral stricture along with bladder neck contracture may appear during follow-up [4]. The incidence of urethral stenosis after TURP is reported to be 2.2 to 9.8% and occurs at this rate in either monopolar or bipolar TURP [1,5]. Many etiologic factors are likely involved, including false passage, mechanical trauma from the large caliber of the instruments utilized, longer duration of surgery and insufficient isolation of the electrical current [6,7].

Management of post-TURP urethral stenosis remains a challenge, and many urologists tend to initially employ endoscopic interventions with an elevated risk of recurrence [7]. Stenosis in the membranous or bulbomembranous urethra has special implications due to its proximity to the urethral sphincter and cavernous nerves [8,9]. Short bulbar and/or membranous stenosis can be treated using a tension-free anastomosis technique, but with well-documented risks of sexual dysfunction and stress urinary incontinence (SUI) [10]. The risk of incontinence after posterior excision urethral and primary anastomosis is also especially high in the case of previous radiation treatment [11,12,13].

Non-transecting techniques using dorsal onlay buccal mucosal graft urethroplasty (DOBMGU) might be preferred with long non-obliterative stenosis and seem to better preserve continence in post-TURP sphincteric membranous urethral strictures [14,15]. To date, there have been no direct comparisons between different techniques for post-TURP membranous stenosis. The aim of this study is to critically analyze a multi-institutional series of DOBMGU for membranous and/or bulbomembranous urethral stenosis in patients with previous TURP and related endoscopic prostate procedures for the treatment of BPO. We hypothesize that this procedure is feasible, provides durable patency and satisfactory patient-reported outcomes with minimal deleterious effects on continence and sexual function.

## 2. Materials and Methods

After obtaining institutional review board approval (A11/20), patients with a history of TURP or other endoscopic prostate procedures for BPO, who underwent DOBMGU for posterior urethral stenosis at participating institutions between 2010 and 2020, were retrospectively reviewed. In accordance with the preferred SIU/ICUD terminology [16], we reviewed cases with “posterior urethral stenosis” to include patients with stenosis of the membranous urethra, extending from the proximal bulbar urethra up to the distal verumontanum. Patients with a history of radiation before or after TURP were included. Patients with bladder neck contracture and urethral stenosis after simple open prostatectomy were excluded. Only patients with a minimum of six months of follow-up were evaluated. 

The primary objective was evaluation of urethral patency and stenosis recurrence along with the incidence of de novo SUI following reconstruction. Secondary objectives included the evaluation of changes in voiding, sexual function and patient satisfaction. Patient demographics, medical history, radiation history, continence status and stenosis length were reviewed. Operative reports were reviewed to identify operative time, complications and estimated blood loss. Perioperative outcomes, stenosis recurrence, pre-operative and post-operative uroflowmetry with post-void residual and validated patient reported outcomes (PROs) such as the Global Response Assessment (GRA), IPSS and SHIM questionnaires were assessed. Questionnaires were taken from the most recent follow-up visit during which they were available. 

### 2.1. Surgical Technique

Dissection of the posterior urethra is performed dorso-laterally, preserving urethral continuity and sparing the bulbar vessels. The urethra is carefully dissected and rotated laterally to spare the striated muscle of the sphincter and the cavernous nerves, the space between the diverging crura is carefully exposed and the dorsal aspect of the urethra is incised longitudinally through the length of the stenosis, with extension approximately 1 cm proximal and distal into the normal urethra [14]. 

Prior to initiating the procedure, passage of a size 4–5 French urethral catheter or a guidewire assists in exposing the membranous urethra by allowing a sharp incision along the guide. No cautery is used in proximity to the crura of the corpora cavernosa, membranous urethra or cavernous nerve mesh. Sharp resection of peri-urethral scar tissue is limited to the area between the 11 and 1 o’clock positions, with careful preservation of the omega-shaped rhabdosphincter. At this point, the lumen is widely exposed and three stitches are passed outside-in, through the proximal end of the urethrotomy at the 11, 12 and 1 o’clock positions, to fix the proximal BMG in place. Careful use of an instrument such as a nasal speculum or gorget probe can assist in the placement of these proximal stitches. An appropriately sized oral graft is harvested and secured to the apices of the urethrotomy with the pre-placed sutures. Quilting sutures are used to fix the graft to the corpora cavernosa and the intercrural space.

### 2.2. Follow-Up

Patients were followed-up with at 3-to-6-month intervals postoperatively, and yearly thereafter. Stenosis recurrence was defined as the need for any intervention, including endoscopic treatments or the need to redo urethroplasty during follow-up. During follow-up in all institutions, patients underwent uroflowmetry and post-void residual measurements and responded to questionnaires. Endoscopic and radiographic follow-up protocols varied between sites, but flexible cystoscopy or radiographic studies were performed with a low threshold for patients inclined to recurrence based on post-operative maximum urinary flow deterioration, worsening IPSS and/or any subjective voiding complaint.

### 2.3. Patient-Reported Outcomes

Patient-reported outcomes were measured by validated questionnaires administered prior to a direct patient interview. The International Prostate Symptom Score with quality-of-life domain (IPSS-QOL) evaluated patient perception of urinary symptoms. The Sexual Health Inventory for Men (SHIM) assessed preoperative and postoperative erectile function. The Global Response Assessment (GRA) determined patients’ satisfaction with the surgery. The GRA is a single query externally validated questionnaire that assesses patients’ impressions of their change in symptoms, and scores range from −3 (markedly worse) to +3 (markedly improved).

### 2.4. Incontinence

Preoperative and postoperative continence status was assessed. Prior to reconstruction, patients were assigned as either preoperatively continent, preoperatively incontinent or unknown. Incontinence was defined as any urine loss. Based on the patient’s clinical history and physical examination, incontinence type was assigned as stress incontinence, urge incontinence or mixed. The “unknown” status was assigned to patients in total urinary retention with suprapubic drainage. The same groups were clinically assessed postoperatively. Only one case with early failure retained a suprapubic tube and an unknown incontinence status after surgery.

### 2.5. Statistical Analysis

Paired *t*-tests compared preoperative and postoperative continuous variables following surgical repair. The Wilcoxon signed-rank test compared the preoperative and postoperative patient-reported survey data. The two-sided Fisher’s exact test was performed for categorical patient-reported data. Factors affecting recurrence-free intervals were evaluated using the Kaplan–Meier analysis method. A multivariate analysis to evaluate factors for stenosis recurrence was performed by stepwise logistic regression with *p* = 0.1 entry and *p* = 0.05 stay criteria. A *p*-value < 0.05 was considered significant. The statistical analysis was performed using Statistical Analysis System 9.4 (SAS Institute Inc., Cary, NC, USA). 

## 3. Results

A total of 107 patients who received intervention from 12 surgeons at nine different institutions between 2010 and 2020 met the inclusion criteria (Table 1). The mean length of stenosis was 3.5 ± 1.8 (0.5–9) cm. There was concomitant involvement of the anterior urethra in 27 patients (25.2%). Prior prostate endoscopic procedures of the participants included 47 patients (44%) with monopolar TURP, 33 (30.8%) with bipolar TURP, 16 (15%) with Greenlight laser, 9 (8.4%) with Holmium laser enucleation and 2 (1.9%) with transurethral bladder neck incision performed 24.1 ± 25 (range 2–192) months prior to urethroplasty. Ten patients (9.3%) also received prior external radiotherapy before urethroplasty. The median time from radiation to urethroplasty was 10 (IQR 10; range 5–50) months. Dilatation before urethroplasty was performed in 72 (67.3%) patients, direct vision internal urethrotomy in 41 (38.3%) and prior anastomotic urethroplasty in 3 (2.8%).

Median surgical time was 152 ± 61 (60–353) mins. No corporal splitting, partial pubectomy, use of flaps, urethral transection or conversion to an abdominoperineal approach was required. Concomitant ancillary procedures for associated anterior urethral stenosis were performed in 19 patients (16.8%) (long dorsal onlay graft as an extension of DOBMGU in 9, separate dorsal onlay graft in 3, separate ventral graft in 1, ASOPA in 1, augmented non-transecting stricturotomy and anastomosis in 1 and ventral meatotomy in 3). Length of hospital stay was 1.4 ± 1.1 (0–9) days and estimated blood loss was 133 ± 119 (10–900) mL. There were seven (6.5%) Clavien–Dindo grade I complications, with two patients presenting with urethral bleeding and five with scrotal hematoma. There were six (5.6%) Clavien–Dindo grade II complications, including five patients with urinary tract infections treated with antibiotics and one patient with a deep venous thrombosis/pulmonary embolism requiring anticoagulation therapy. There was one (0.9%) Clavien–Dindo grade III complication requiring operative intervention for buccal graft harvest site rebleeding.

### 3.1. Patency Outcomes

**Figure 1:**
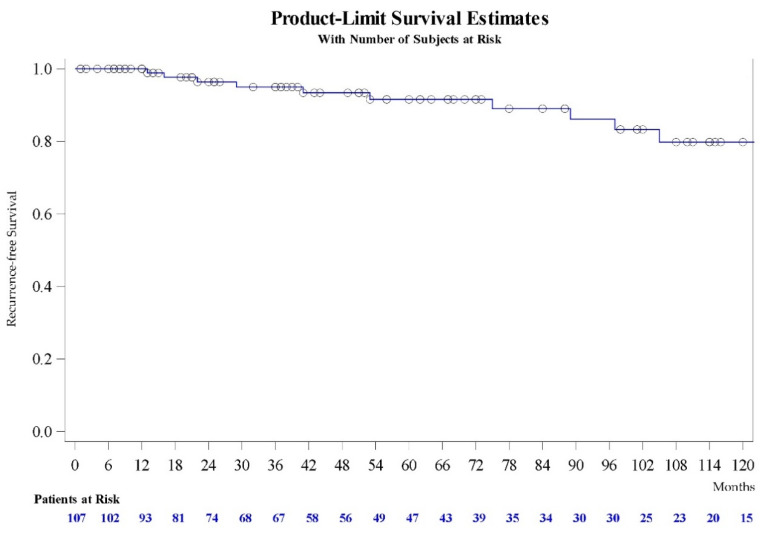
Kaplan–Meier curve of recurrence free survival of urethral stenosis after bulbomembranous dorsal onlay buccal mucosa graft urethroplasty (DOBMGU).

At a mean of 59.3 ± 45.1 months follow-up (range 6–148), re-stenosis occurred in 10 patients (9.35%). Kaplan–Meier survival revealed that 91% (95% CI 81.9–96.2) of patients were free of recurrence at 5 years and 79.8% (95% CI 64–89.2) at 10 years (Figure 1). Univariate analysis revealed diabetes, smoking, monopolar TURP, associated radiation, prior dilatation, prior DVIU, stricture length and postoperative complications within 90 days could be associated with stenosis recurrence during follow-up (Table 2). These factors were included in the regression model for recurrence. Multivariate analysis confirmed postoperative complications (OR 12.5 (95% CI 1.9–83.5); *p* = 0.009), associated radiation (OR 8.3 (95% CI 1.49–46.34); *p* = 0.016) and ≥2 dilatations before urethroplasty (OR 7.2 (95% CI 1.2–43.6); *p* = 0.032) as independent predictors of recurrence. 

### 3.2. Clinical Outcomes (Uroflow, PVR, IPSS)

IPSS, IPSS-QoL, post void residual and Qmax were evaluated pre- and postoperatively in 99 cases (92.5%). Qmax improved from 6.2 ± 3.1 (mean ± SD) to 16.8 ± 5.4 cc/s (*p* < 0.001), PVR from 128 ± 83 to 60 ± 80 cc (*p* < 0.001), total IPSS from 20 ± 5.6 to 7.7 ± 6 (*p* < 0.001) and IPSS-QoL from 4.4 ± 1 to 1.7 ± 1.4 (*p* < 0.001). The proportion of patients with severe urinary symptoms (IPSS 20 to 35) fell from 52.5% (52/99) to 6% (6/99) (*p* = 0.006). 

### 3.3. Continence Outcomes

Before surgery, 87 patients (81.3%) were continent and used no pads, 16 (15%) were incontinent (4 stress, 9 urge and 3 mixed incontinence) and in 4 cases (3.7%), continent status was unknown due to a complete obstruction. Only 1 patient (0.9%) developed de novo SUI following DOBMGU. After surgery, 94 patients (87.9%) were continent, 12 (11.2%) were incontinent (7 stress, 4 urge and 1 mixed) and in 1 case (0.9%), continent status was unknown due to obstruction. 

### 3.4. Sexual Function Outcomes

SHIM and other sexual aspects such as penile pain and weak ejaculation were evaluated pre- and postoperatively with data available in 87 patients (81.3%). Mean SHIM value improved from 11.5 ± 6.3 to 11.7 ± 7 (*p* = 0.028). Of 22 patients with a SHIM score of ≥17 (mild ED or no dysfunction) preoperatively, 2 (9%) scored ≤11 (severe to moderate ED) and 4 (18.2%) scored from 12–16 (mild to moderate ED) postoperatively. Conversely, of 22 patients with a SHIM score of ≤11 (severe to moderate ED), 9 (22%) improved to 12–16 and 2 (4.9%) to ≥17 postoperatively. There was no variation in the percentage of patients with penile pain (9.5% preoperatively vs. 3.8% postoperatively; *p* = 0.1) but the percentage of patients with postoperative weak ejaculation decreased from 24.5% preoperatively to 3.8% after surgery (*p* < 0.0001). However, narrative data on how many patients really could ejaculate before urethroplasty, and among them how they ejaculate after the urethral repair, are not available.

### 3.5. Global Response Assessment Outcomes

GRA outcomes were available in all 107 patients, of whom 97 (90.7%) assessed their situation as better than before (GRA + 1 or higher), 87 cases (81.3%) self-defined as much better than before (GRA of + 2 or + 3), and 70 (65.4%) registered themselves as “very much better than before” (GRA + 3). A regression analysis was performed to evaluate factors associated with the best patient perception, and the absence of radiation was the only independent factor associated with a patient definition of GRA + 3 (0R 0.12 (95% CI 0.02–0.62); *p* = 0.011). 

## 4. Discussion

Due to the proximity of the rhabdosphincter and cavernous nerves to the membranous urethra, reconstruction of membranous stenosis implies a risk of urinary incontinence and erectile dysfunction, and this concern is greater in patients with previous TURP in which the bladder neck and the internal sphincteric mechanism may be disrupted [17,18]. This could explain the high rates of incontinence observed with excision and primary anastomosis in classic studies facing sphincteric strictures, especially after pelvic fracture and radiation [10,13,19,20].

There is increasing evidence that membranous dorsal onlay urethroplasty, not only in the context of radiation but also after TURP, does not compromise continence or erectile function in most patients [14,21,22]. The multi-institutional experience we present reveals a limited failure rate (9.35%) that is very comparable to intrasphincteric anastomotic urethroplasty after BPO surgery (3.9–10%) [17,23]. However, the mean stricture length of the patients treated with DOBMGU was longer (3.5 cm) and the proportion of de novo incontinence lower (0.9%) compared to intrasphincteric anastomosis (1.5–2.6 cm and 7.8–15%, respectively), and these studies excluded previous radiation and incontinence before urethroplasty [17,23]. Our study includes previous radiation and concomitant need of anterior urethroplasty and gives a better definition of the different types of incontinence both pre- and postoperatively. Additionally, we report favorable SHIM scores, other sexual symptoms (penile pain, ejaculation) and PROMs (both IPSS-QoL and GRA) following repair. Multi-institutional retrospective studies have the advantage of collecting a large number of patients, but at the same time suffer from the limitation of technical heterogeneity This study is also limited by its retrospective non-comparative design, but suggests that DOBMGU is a safe and efficacious technique to treat post-TURP bulbomembranous stenosis.

**Figure 2:**
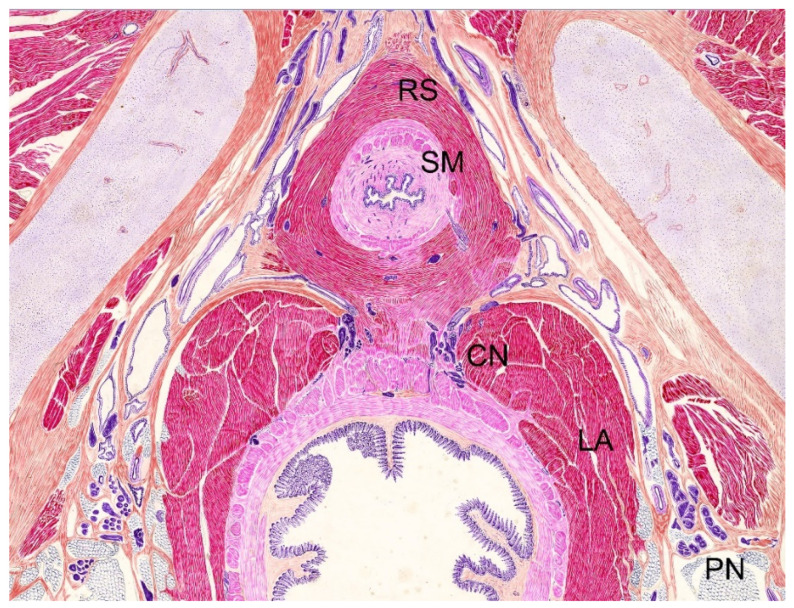
Indian ink on paper drawing from histotopographic 40-μm transverse section of a male fetus from the Salvador Gil Vernet collection (courtesy of Jose Maria Gil-Vernet Sedó) revealing the omega-shaped circular bundles of rhabdosphincter (RS), inner circular bundles of smooth muscle (SM), levator ani (LA), cavernous nerve (CN) and pudendal nerve (PN).

In fact, the European Guidelines on urethral stricture suggest conducting bulbomembranous urethral stenosis after TURP similar to bulbar strictures, either by excision and primary anastomosis or augmentation urethroplasty, with a graft according to the length and tightness of the stricture, while recognizing the high risk of incontinence when reconstruction takes place in the proximity of the external sphincter after damaging the bladder neck during BPO surgery [24]. However, surgical refinement allows careful sphincteric preservation and removal of the fibrous tissue using a dorsal urethrotomy. Erectile impairment after urethroplasty is largely mediated through cavernous nerve injury [8,25]. Cavernous nerves could be better preserved by a dorsal approach than a ventral or circumferential one, as nerve fibers stand in the 5 and 7 o’clock positions (Figure 2). Accordingly, Hinata and colleagues have previously described a U-shaped cavernous nerve mesh outside the periprostatic region which would spare the 12 o’clock position [26]. 

Anatomic studies regarding the preservation of erectile function after bulboprostatic anastomosis led to better characterization of the cavernous neurovascular bundle paths, and also to the discovery of a delicate sheath of connective tissue between the wall of the membranous urethra and the surrounding circular fibers of the external sphincter [25,27]. This sheath can be used as a surgical plane to separate the urethral wall from the sphincter. The circular muscle fibers of the external sphincter can be reflected from the sheath until the underlying urethral wall is identified and dissected [8]. This allows intra-sphincteric dissection to perform bulboprostatic anastomosis or intrasphincteric anastomotic urethroplasty, as defined by Gómez et al., preserving continence and sparing the bulbar vessels [17]. This technique is optimal in cases with short and occlusive stenosis of the membranous urethra, especially in men after BPO surgery. However, cases with longer stenosis may benefit more from augmentation techniques.

It has been suggested and assumed, without evidence, that ventral onlay graft techniques in proximal bulbar and membranous urethra better preserve the sphincter and cavernous mesh than dorsal onlay grafts [9,28], but membranous dorsal and ventral approaches have not been compared to date. A recent retrospective study including 69 patients with post-TURP stenosis in the area of the distal sphincter confirms that careful ventral onlay graft urethroplasty is a suitable technique after BPO transurethral surgery, demonstrating 84% patency success at a median follow-up of 52 months and a 4.3% incidence of de novo incontinence [15]. The risks of recurrence and of de novo incontinence increase to 28.9% and 10.5%, respectively, when a bulbomembranous ventral onlay graft is performed after radiation [29]. In this sense, a recent multi-institutional assessment of dorsal onlay urethroplasty for post-radiation urethral stenosis revealed a 17.7% risk of recurrence and an 8.1% risk of de novo incontinence [21].

Posterior urethral stenosis repair following surgical endoscopic procedures for BPO is a very challenging situation. This series has been gathered from a retrospective evaluation of patients from nine academic institutions who were operated on and followed-up with over a long period. However, the number of cases per institution per year is not lineal, as not all centers adopted this technique at the same time and the volume of cases from each institution is variable. Our combined experience, including both surgical outcomes and PROs, suggests DOBMG urethroplasty can be a feasible option. However, important limitations should be recognized. First of all, the absence of a standardized preoperative protocol in these types of studies limits the accurate evaluation of whether a bulbomembranous stenosis is truly trans-sphincteric or not, especially in cases where urethroscopy does not allow access to the area. Antegrade cystoscopy and urethrogram may add limited information to help solve the issue. The description of the operative report is, although inaccurate, the most reliable observation to categorize the stenosis. Secondly, the definition of failure as the need for re-intervention may overestimate success. Finally, but not less importantly, PROs should be better estimated in prospective studies to avoid any likely bias. 

This perplexing condition may be better treated and followed-up with in highly specialized centers to offer the maximum possibility of continence and erectile function preservation to patients. The classic option to treat bulbomembranous stenosis has been excision and primary anastomoses. However, in cases with previous TURP, evidence is accumulating in favor of intrasphincteric anastomotic urethroplasty and onlay graft techniques. The experience we report suggests dorsal BMG urethroplasty is efficacious and safe. Of course, prospective controlled trials comparing different techniques are needed to say which technique is most advantageous over the others. Therefore, further investigation is needed on the subject.

## 5. Conclusions

DOBMGU is a safe and efficacious technique to treat post-TURP bulbomembranous stenosis, with long-term durability. Patients at risk of recurrence are those with postoperative complications, a radiation history and ≥2 dilatations prior to urethroplasty. This non-transecting urethral reconstruction spares the bulbar vessels, cavernous nerves and striated sphincter, while further conferring a low risk of de novo SUI in prior TURP recipients. Further work is needed to compare this technique with other surgical approaches, such as ventral onlay, intrasphincteric anastomotic urethroplasty and classical excision and primary anastomosis, used to treat this challenging patient population.

## Figures and Tables

**Table 1:** Characteristics of the patients included in the study (*n* = 107). * Mean ± SD; ^1^ HT/CVD, hypertension and/or cardiovascular disease; ^2^ Smoking includes tobacco chewing habit; ^3^ TURP, transurethral resection of the prostate; ^4^ DVIU, direct vision internal urethrotomy.

Variables	*n* (%)
Age, year *	69.1 ± 9.5
Body mass index, kg/m^2^ *	27.7 ± 5.1
HT/CVD ^1^	44 (41.1)
Diabetes mellitus	32 (29.9)
Active smoking ^2^	29 (27.1)
Race	
Caucasian	81 (75.7)
Black	8 (7.5)
Asian	5 (4.7)
Latin	13 (12.1)
Type of TURP ^3^	
Monopolar TURP	47 (44.3)
Bipolar TURP	33 (31.1)
Greenlight laser	16 (15.1)
Holmium enucleation	9 (8.5)
Bladder neck incision	2 (1.9)
Time from TURP to urethroplasty, month *	24.1 ± 25
Prior dilatation	
None	35 (32.7)
One	32 (29.9)
Two or more (up to 20)	40 (37.4)
Prior DVIU ^4^	
None	66 (61.7)
One	27 (25.2)
Two or more (up to 4)	14 (13.1)
Prior urethroplasty	3 (2.8)
Stricture length, cm *	3.5 ± 1.85
Concomitant involvement of anterior urethra	27 (25.2)
Use of ancillary techniques	19 (16.8%)
Follow-up since urethroplasty, month *	59.3 ± 45.1
Stenosis recurrence during follow-up	10 (9.35)

**Table 2:** Association between stenosis recurrence and clinical features. Variables with statistical significance (*p* < 0.05) are highlighted in bold; variables with *p* < 0.1 were considered for the stepwise model regression analysis (*). ^1^ HT/CVD, hypertension and/or cardiovascular disease; ^2^ TURP, transurethral resection of the prostate; ^3^ DVIU, direct vision internal urethrotomy; ^4^ According to Clavien–Dindo classification within 90 postoperative days.

Variables	*n*	Stenosis Recurrence		*p* Value
		% With	% Without	
Patient age	<70 years (*n* = 55)	-	66.7	0.339
	≥70 years (*n* = 52)	50	33.3	
HT/CVD ^1^	No (*n* = 63)	58.8	60	0.94
	Yes (*n* = 44)	41.2	40	
Diabetes Mellitus	No (*n* = 75)	73.2	40	**0.029** *
	Yes (*n* = 32)	26.8	60	
Smoking habit	No (*n* = 78)	75.3	50	0.087 *
	Yes (*n* = 29)	24.7	50	
Type of TURP ^2^	Monopolar TURP (*n* = 47)	25.9	63.6	**0.049** *
	Bipolar TURP (*n* = 33)	14.7	27.3	
	Greenlight laser (*n* = 16)	0	0	
	Holmium laser (*n* = 9)	9.4	0	
	Bladder neck incision (*n* = 2)	1	9.1	
Radiation	No (*n* = 97)	93.8	60	**0.006** *
	Yes (*n* = 10)	6.2	40	
Prior dilatation	None or 1 (*n* = 67)	66	30	**0.038** *
	More than 1 (*n* = 40)	34	70	
Prior DVIU ^3^	No (*n* = 41)	36.1	60	0.069 *
	Yes (*n* = 66)	63.9	40	
Prior urethroplasty	No (*n* = 104)	96.9	100	1
	Yes (*n* = 3)	3.1	0	
Stricture length	<4 cm (*n* = 77)	75.3	40	**0.027** *
	≥4 cm (*n* = 30)	24.7	60	
Associated anterior stenosis	No (*n* = 80)	75.3	70	0.71
	Yes (*n* = 27)	24.7	30	
Postoperative complications ^4^	No (*n* = 99)	89.7	60	**0.025** *
	Yes (*n* = 14)	10.3	40	

## Data Availability

Data supporting reported results will be provided by the correspondence author upon reasonable request.

## References

[1] Rassweiler J., Teber D., Kuntz R., Hofmann R. (2006). Complications of Transurethral Resection of the Prostate (TURP)—Incidence, Management, and Prevention. Eur. Urol..

[2] Mamoulakis C., Ubbink D.T., de la Rosette J.J. (2009). Bipolar versus Monopolar Transurethral Resection of the Prostate: A Systematic Review and Meta-analysis of Randomized Controlled Trials. Eur. Urol..

[3] Gravas S., Cornu J.N., Gacci M., Gratzke C., Herrmann T.R.W., Mamoulakis C., Rieken M., Speakman M.J., Tikkinen K.A.O., Karavitakis M. (2019). EAU Guidelines on Management of Non-Neurogenic Male Lowr Urinary Tract Symptoms (LUTS), Including Benign Prostatic Obstruction (BPO).

[4] Tascı A.I., Ilbey Y.O., Tugcu V., Cicekler O., Cevik C., Zoroglu F. (2011). Transurethral resection of the prostate with monopolar resecto-scope: Single-surgeon experience and long-term results of after 3589 procedures. Urology.

[5] Tang Y., Li J., Pu C., Bai Y., Yuan H., Wei Q., Han P. (2014). Bipolar Transurethral Resection Versus Monopolar Transurethral Resection for Benign Prostatic Hypertrophy: A Systematic Review and Meta-Analysis. J. Endourol..

[6] Komura K., Inamoto T., Takai T., Uchimoto T., Saito K., Tanda N., Minami K., Oide R., Uehara H., Takahara K. (2015). Incidence of urethral stricture after bipolar transurethral resection of the prostate using TURis: Results from a randomised trial. BJU Int..

[7] Wang J.-W., Man L.-B. (2020). Transurethral resection of the prostate stricture management. Asian J. Androl..

[8] Angulo J.C., Gómez R.G., Nikolavsky D. (2018). Reconstruction of Membranous Urethral Strictures. Curr. Urol. Rep..

[9] Kulkarni S.B., Joglekar O., Alkandari M., Joshi P.M. (2018). Management of post TURP strictures. World J. Urol..

[10] Mundy A.R. (1989). The Treatment of Sphincter Strictures. BJU Int..

[11] Hofer M.D., Zhao L.C., Morey A.F., Scott J.F., Chang A.J., Brandes S.B., Gonzalez C.M. (2014). Outcomes after Urethroplasty for Radiotherapy Induced Bulbomembranous Urethral Stricture Disease. J. Urol..

[12] Rourke K., Kinnaird A., Zorn J. (2016). Observations and outcomes of urethroplasty for bulbomembranous stenosis after radiation therapy for prostate cancer. World J. Urol..

[13] Chung P.H., Esposito P., Wessells H., Voelzke B.B. (2018). Incidence of stress urinary incontinence after posterior urethroplasty for ra-diation-induced urethral strictures. Urology.

[14] Gimbernat H., Arance I., Redondo C., Meilan E., Andres G., Angulo J.C. (2014). Treatment for long bulbar urethral strictures with membranous involvement using urethroplasty with oral mucosa graft. Actas Urol. Esp..

[15] Barbagli G., Kulkarni S.B., Joshi P.M., Nikolavsky D., Montorsi F., Sansalone S., Loreto C., Lazzeri M. (2019). Repair of sphincter urethral strictures pre-serving urinary continence: Surgical technique and outcomes. World J. Urol..

[16] Latini J.M., McAninch J.W., Brandes S.B., Chung J.Y., Rosenstein D. (2014). SIU/ICUD Consultation on Urethral Strictures: Epidemiology, Etiology, Anatomy, and Nomenclature of Urethral Stenoses, Strictures, and Pelvic Fracture Urethral Disruption Injuries. Urology.

[17] Gómez R.G., Velarde L.G., Campos R.A., Saavedra A.A., Delgado E.J., Santucci R.A., Scarberry K.A. (2020). Intrasphincteric anastomotic urethroplasty allows preservation of continence in men with bulbomembranous urethral strictures following benign prostatic hyperplasia surgery. World J. Urol..

[18] Gomez R.G., Scarberry K. (2018). Anatomy and techniques in posterior urethroplasty. Transl. Androl. Urol..

[19] Mundy A. (1996). Urethroplasty for posterior urethral strictures. BJU Int..

[20] Meeks J.J., Brandes S.B., Morey A.F., Thom M., Mehdiratta N., Valadez C., Granieri M.A., Gonzalez C.M. (2011). Urethroplasty for Radiotherapy Induced Bulbomembranous Strictures: A Multi-Institutional Experience. J. Urol..

[21] Policastro C.G., Simhan J., Martins F.E., Lumen N., Venkatesan K., Angulo J.C., Gupta S., Rusilko P., Pérez E.A.R., Redger K. (2020). A multi-institutional critical assessment of dorsal onlay urethroplasty for post-radiation urethral stenosis. World J. Urol..

[22] Blakely S., Caza T., Landas S., Nikolavsky D. (2016). Dorsal Onlay Urethroplasty for Membranous Urethral Strictures: Urinary and Erectile Functional Outcomes. J. Urol..

[23] Favre G.A., Alfieri A.G., Gil V.S.A., Tobia I., Giudice C.R. (2021). Bulbomembranous Urethral Strictures Repair After Surgical Treatment of Benign Prostatic Hyperplasia. Experience From a Latin American Referral Centre. Urology.

[24] Lumen N., Campos-Juanatey F., Dimitropoulos K., Greenwell T., Martins F.E., Osman N., Riechardt S., Waterloos M., Barratt R., Chan G. EAU Guidelines on Urethral Stricture. https://uroweb.org/wp-content/uploads/EAU-Guidelines-on-Urethral-Strictures1-2021.pdf.

[25] Al-Rifaei M.A., Zaghloul S., Al-Rifaei A.M. (2005). Bulboprostatic anastomotic urethroplasty with preservation of potency: Anatomical study, operative approach and clinical results. Scand. J. Urol. Nephrol..

[26] Hinata N., Murakami G., Miyake H., Abe S., Fujisawa M. (2015). Histological Study of the Cavernous Nerve Mesh Outside the Periprostatic Region: Anatomical Basis for Erectile Function after Nonnerve Sparing Radical Prostatectomy. J. Urol..

[27] Dalpiaz O., Mitterberger M., Kerschbaumer A., Pinggera G.-M., Bartsch G., Strasser H. (2008). Anatomical approach for surgery of the male posterior urethra. BJU Int..

[28] Barbagli G., Montorsi F., Guazzoni G.F., Larcher A., Fossati N., Sansalone S., Romano G., Buffi N., Lazzeri M. (2013). Ventral Oral Mucosal Onlay Graft Urethroplasty in Nontraumatic Bulbar Urethral Strictures: Surgical Technique and Multivariable Analysis of Results in 214 Patients. Eur. Urol..

[29] Ahyai S.A., Schmid M., Kuhl M., Kluth L.A., Soave A., Riechardt S., Chun F.K.H., Engel O., Fisch M., Dahlem R. (2015). Outcomes of ventral onlay buccal mucosa graft urethroplasty in patients after radiotherapy. J. Urol..

